# Effects of local structure of Ce^3+^ ions on luminescent properties of Y_3_Al_5_O_12_:Ce nanoparticles

**DOI:** 10.1038/srep22238

**Published:** 2016-03-03

**Authors:** Xiaowu He, Xiaofang Liu, Rongfeng Li, Bai Yang, Kaili Yu, Min Zeng, Ronghai Yu

**Affiliations:** 1 School of Materials Science and Engineering, Beihang University, Beijing, 100191, P. R. China

## Abstract

Ce^3+^-doped yttrium aluminum garnet (YAG:Ce) nanocrystals were successfully synthesized via a facile sol-gel method. Multiple characterization techniques were employed to study the structure, morphology, composition and photoluminescence properties of YAG:Ce nanophosphors. The YAG:Ce_0.0055_ sintered at 1030 °C exhibited a typical 5*d*^1^-4*f*^1^ emission band with the maximum peak located at 525 nm, and owned a short fluorescence lifetime *τ*_1_ (~28 ns) and a long fluorescence lifetime *τ*_2_ (~94 ns). Calcination temperature and Ce^3+^ doping concentration have significant effects on the photoluminescence properties of the YAG:Ce nanophosphors. The emission intensity was enhanced as the calcination temperature increased from 830 to 1030 °C, but decreased dramatically with the increase of Ce^3+^ doping concentration from 0.55 to 5.50 at.% due to the concentration quenching. By optimizing the synthesized condition, the strongest photoluminescence emission intensity was achieved at 1030 °C with Ce^3+^ concentration of 0.55 at.%.

The energy-efficient white light-emitting diode (WLED) has been widely applied as a solid-state optical source in various fields such as general lighting, car lighting backlighting sources, apparatus display screen and so on^1^,^2^,^3^,^4^,^5^,^6^. Generally, white light can be produced through three approaches: a blue LED combining with yellow phosphors, an ultraviolet LED matching with mixture of yellow (or red and green) phosphors and blue phosphors, and a device consisting of red, green and blue LEDs^7^,^8^,^9^,^10^. Among them, the blue LED plus yellow phosphor system is prior selected as the commercial WLEDs due to its facile fabrication and low cost, as well as nice colour-rendering index (CRI)^10^. Since the YAG:Ce phosphor was firstly used in displays by Blasse *et al.*^11^ in 1967, it widely acted as a classical down-conversion material because of its high quantum efficiency, high refractive index, good mechanical strength, outstanding chemical and thermal stability.

Traditionally, YAG:Ce phosphors are easily agglomerated via solid-state reaction at high temperature (>1500 °C) and thus post-milling is required, which would cause the disadvantages of oversize grains and incorporation of impurities^11^,^12^,^13^. Although molten salt method can efficiently reduce calcination temperature, the postprocessing becomes more complex^14^. In order to overcome the above shortcomings, wet-chemical synthetic approaches have been developed for fabricating pure and homogeneous YAG:Ce phosphors. For instance, He *et al.*^15^,^16^ obtained YAG:Ce phosphors by spray pyrolysis method and could randomly control the grain size and composition of product particles with nano/microsphere morphology. Murai *et al.*^17^ synthesized uniform Y_3_Al_5_O_12_:Ce nanocrystals with a particle size less than 50 nm by sol-gel route. Jiao *et al.*^18^ successfully synthesized pure nano-scale Ce-doped YAG phosphors via LiF assisted sol-gel method at a lower temperature of 540 °C. Thus, sol-gel method, which is simple and controllable, is widely used in fabrication of YAG:Ce phosphors. The blue absorption and yellow emission of the Ce^3+^-doped YAG phosphors, which are attributed to 4*f*^ 1^ → 4*f*^  0^5*d*^1^ and 4*f*^  0^5*d*^1^ → 4*f *^1^ transitions of Ce^3+^ ion^19^, respectively. The participation of 5*d* energy levels makes the transitions be sensitive to the crystal field environment and the site symmetry of the host lattice, i.e. local structure of Ce_Y_ substitution. Therefore, both of the emission intensity and wavelength depend on the local structure and distribution of the Ce_Y_ in YAG:Ce phosphors^20^,^21^. George *et al.*^22^ studied the local environment of Ce^3+^ activators in Y_3_Al_5_O_12_:Ce phosphors and found that the random distribution of Ce^3+^ ions in YAG lattice could result in the slight expansion of YAG unit cell, which played critical roles in enhancing quantum yield and preventing from photoluminescence quenching. Belén *et al.*^23^ studied the interplay between the Ce_Y_ and Y_Al_-Al_Y_ in YAG by using the first-principles calculations and found that the presence of Y_Al_-Al_Y_ could cause a strongly anisotropic expansion of the atomistic structure around the Ce_Y_ impurities and decrease the effective ligand-field splitting of the 5*d*_1_ manifold, leading to the blue-shifts the two lowest Ce^3+^ 4f–5d transitions. Although the photoluminescence properties of Ce^3+^ ions in garnet structure were investigated^24^, the detailed relationship between the photoluminescence properties and the local structures as well as the distributions of activators in Ce^3+^-doped YAG phosphors have been rarely studied experimentally.

In this work, a series of YAG:Ce nanocrystalline phosphors were prepared by sol-gel method^25^ in terms of its advantages of uniform distribution of Ce^3+^ ions, fast reaction rate and low calcination temperature. The composition, structure and morphology of the YAG:Ce_*x*_ nanophosphors were well tuned by changing the Ce^3+^ concentration and the calcination temperature, aiming to enhance their emission intensity. Using multiple characterization techniques, we systematically clarified the intrinsic relationship among composition, structure and property.

## Results and Discussion

### Phase formation and crystal structure

**Figure 1:**
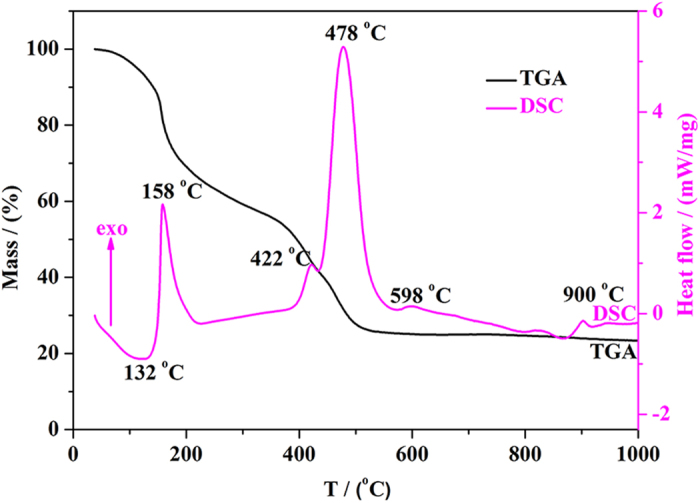
DSC-TGA profiles for the thermal variation in the precursor of sample-1.

Figure 1 shows the DSC-TGA curves corresponding to the agglomeration process of YAG:Ce sample-1 from precursors to phosphors. The DSC curve exhibits the first endothermic peak at 132 °C, which originates from the evaporation of residual water. The strong exothermic peak at 158 °C is probably ascribed to the elimination of chelating agent molecules. Moreover, two intense exothermic peaks around 422 and 478 °C could be attributed to the pyrolysis of organic compounds and degradation of intermediate species formed during the gelation process, respectively^26^. From the TGA curve, it is found that a considerable weight loss in stages occurred with increasing temperature before 598 °C and the total weight loss was ~75 wt.%. However, the TGA curve almost keeps constant when the sintering temperature is above 598 °C. This phenomenon indicates the beginning of crystallization^21^, which is further confirmed by the appearance of exothermic peak at 900 °C. Thus, the crystallization temperature of YAG:Ce phosphors synthesized via sol-gel method is probably in the range of 830–900 °C.

**Figure 2:**
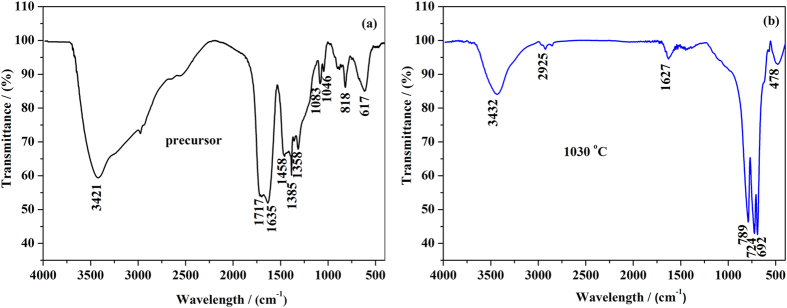
FT-IR spectra: (**a**) the precursor of YAG:Ce sample-1 (100 °C, 24 h) and (**b**) the powder of YAG:Ce sample-1 (1030 °C, 3 h).

The FT-IR spectrum of YAG:Ce sample-1 precursor is shown in Fig. 2(a). The intense and broad envelope of band at ~3421 cm^−1^ is due to the O-H vibration caused by H_2_O in the sample^27^. The absorption bands at 1635 and 1458 cm^−1^ should be ascribed to stretching vibrations of the -COO- groups^20^. Comparing with the standard citric acid (at 1717 cm^−1^), these -COO- groups shift to lower frequency because of the coordination of -COO- groups and metal ions. The strong absorption bands at 1385 cm^−1^ originate from the surface-adsorbed nitrate groups^20^. The absorption bands at 1083 and 1046 cm^−1^ could be attributed to O-H bend^20^. Moreover, the several broad bands observed within the 550–850 cm^−1^ region of the IR spectrum correspond to M-O bonds (M = Y or Al) vibrations in the precursor^28^. The FT-IR spectrum of YAG:Ce sample-1 is presented in Fig. 2(b). The intense and broad envelope of bands at ~3432 cm^−1^ is due to the stretching vibration of adsorbed H_2_O. The peaks at ~789 and ~692 cm^−1^ are the characteristic vibrations of Al-O (metal-oxygen) while the peaks at ~724 and ~478 cm^−1^ represent the Y-O (metal-oxygen) vibrations^26^. The presence of intensive vibration peaks of Y-O and Al-O suggests that the YAG:Ce sample-1 was crystallized at 1030 °C.

**Figure 3:**
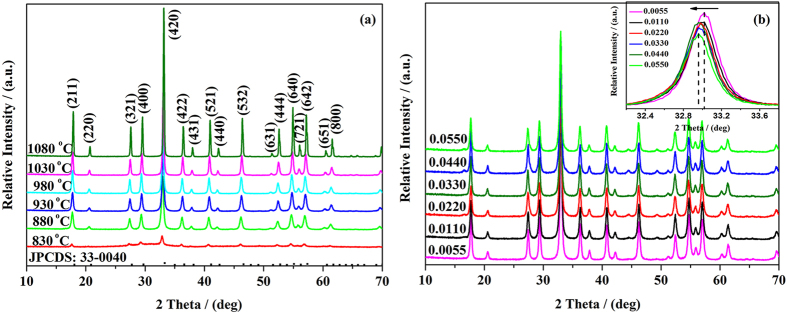
The XRD patterns of (**a**) YAG:Ce sample-1 sintered at different temperatures for 3 h and (**b**) YAG:Ce samples sintered at 1030 °C for 3 h with different Ce^3+^ concentration.

Figure 3(a) displays the XRD patterns of the YAG:Ce sample-1 sintered at different temperatures. The positions of the diffraction peaks for the six samples are similar and consistent with the standard cubic structure of Y_3_Al_5_O_12_ (JCPDS: 33–0040) and space group Ia-3d (230)^29^. It implies that the pure YAG phase can be successfully obtained at a temperature as low as 830 °C and no other crystalline phases such as Y_4_Al_12_O_9_ (YAM) or YAlO_3_ (YAP) form. Therefore, the crystallization temperature can be effectively decreased through the sol-gel method and the lowest crystallization temperature is found to be 830 °C in our work. With the increase of calcination temperature, the diffraction peaks of YAG:Ce sample-1 become strong and sharp, demonstrating the increase of crystallinity. Meanwhile, the crystalline size can be estimated by Scherrer’s formula. It was clear that the crystalline mean grain size increases with the increase of sintering temperature as shown in Table 1. Figure 3(b) shows the XRD patterns of YAG:Ce samples with different Ce^3+^-doped concentration sintered at 1030 °C. All the diffraction peaks can be indexed as YAG phase and no impurity peaks are detected, which reveals that the incorporated Ce^3+^ ions do not change the crystalline structure of YAG. Nevertheless, the peak positions of diffraction peaks shift towards low angle region due to the larger ionic radius of Ce^3+^ ion (1.07 Å) than that of Y^3+^ ion (0.92 Å). It is well known that pure phase YAG:Ce phosphors with high crystallinity is critical to obtain large-powder WLEDs with high efficiency^30^,^31^. Hence, it is expected that the synthesized Ce^3+^-doped YAG phosphor possesses good photoluminescence property.

**Figure 4:**
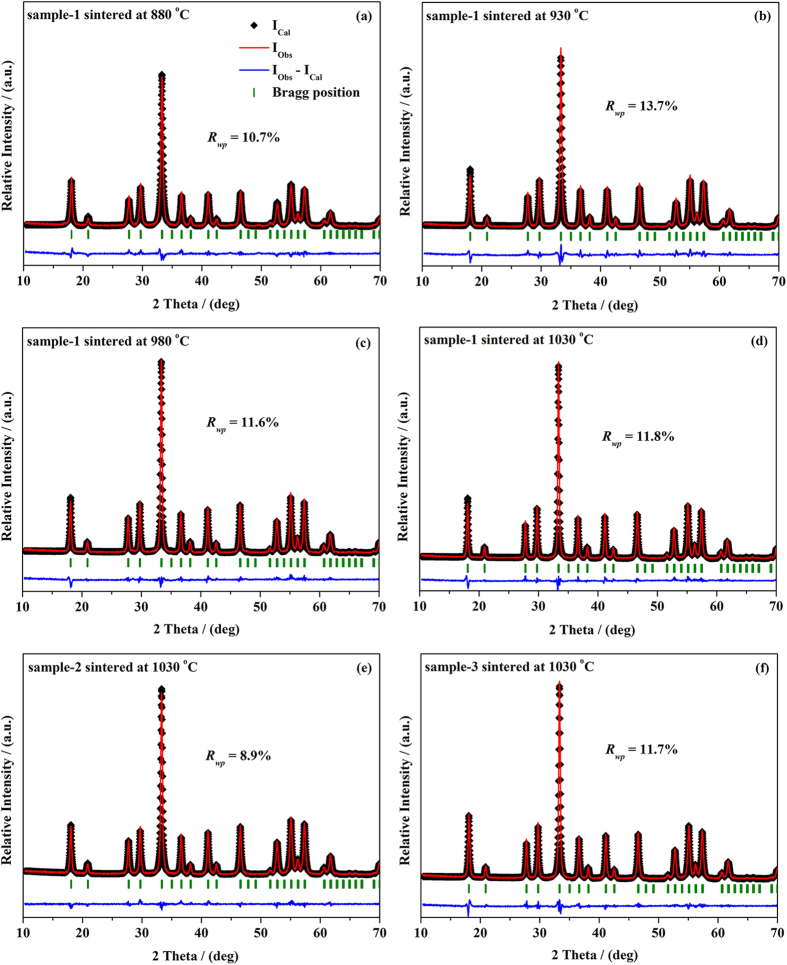
Rietveld refinement. (**a–d**) YAG:Ce sample-1 sintered at different temperatures for 3 h and (**d–f**) YAG:Ce sample-1, -2 and -3 sintered at 1030 °C for 3 h with different Ce^3+^ concentration.

To confirm the formation of YAG:Ce phosphors in the cubic phase, Rietveld refinement was performed on the XRD patterns using the Fullprof 2K software. Figure 4 presents the Rietveld refinement results for the YAG:Ce sample-1 sintered at different temperatures and the YAG:Ce sample-1, -2 and -3 with different Ce^3+^ doped concentration, respectively, and simultaneously the crystallographic data are summarized in Table 1. It can be seen from Fig. 4(a–d) that the unit cell parameters (*a*) decreased with increasing calcination temperature, keeping accordance with the shift of the position of diffraction peak to larger angle in the XRD patterns^20^. Meanwhile, the results from Fig. 4(d–f) show that the unit lattice parameters (*a*) become larger with increasing Ce^3+^ concentration due to the larger Ce^3+^ ionic radius than that of Y^3+^ ion. It suggests that the unit cell parameters of YAG:Ce nanocrystalline could be expanded with increasing the amount of Ce^3+^ ions into Y lattice sites gradually.

**Figure 5:**
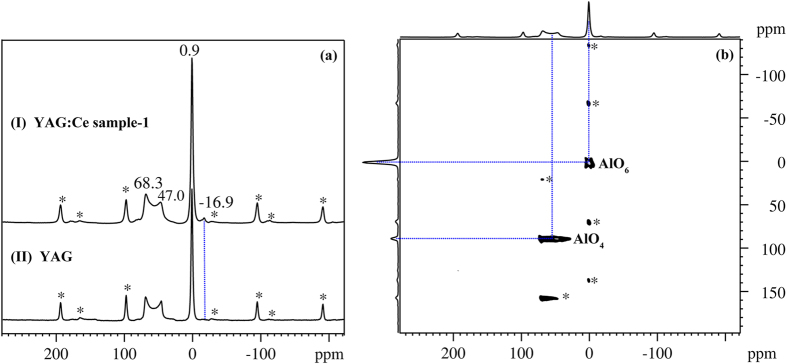
(**a**) ^27^Al MAS NMR spectra of YAG and YAG:Ce sample-1 and (**b**) ^27^Al 3Q MAS NMR spectrum of YAG:Ce sample-1 sintered at 1030 °C for 3 h. Spin rate was 10 kHz and the asterisks stand for spinning bands.

The high-resolution magic angle spinning (MAS) ^27^Al NMR spectroscopy was carried out for identifying the different phases and coordination states of Al sites in Al-containing materials because of the high sensitivity of ^27^Al NMR chemical shift towards the local chemical environment of Al nucleus, especial for the material with short range order where powder XRD provided little information about the Al distribution^32^,^33^. Figure 5(a) provides the ^27^Al MAS NMR spectra of YAG (down) and YAG:Ce sample-1 (up) sintered at 1030 °C for 3 h. The sharp and narrow line at 0.9 ppm observed for both of YAG and YAG:Ce sample-1 could be assigned to the octahedral AlO_6_ species^32^. Another weak signal at −16.9 ppm is located within chemical shift range of AlO_6_ species as well^33^,^34^. From the relative intensity of these two peaks, it is concluded that the weak line at −16.9 ppm arises from the AlO_6_ units suffering from the chemical influence of the tiny amount of Ce^3+^ ions, keeping accordance with the previous literature^22^. Its chemical shift to higher field relative to typical AlO_6_ species (0.9 ppm) was caused by the adjacent Ce^3+^ whose unpaired electrons in the 4f shell influence the local magnetic field of adjacent Al nuclei, which is further supported by the absence of peak at −16.9 ppm in the spectrum of the YAG without Ce^3+^ ions doping (Fig. 5(a) down). Additionally, the other broad peak appeared at 47.0–68.3 ppm can be attributed to the second-order quadrupole line shape of hexahedral AlO_4_ species due to the lower symmetry of the crystallographic site with respect to the octahedral AlO_6_ units^34^,^35^. And it was confirmed further by the high-resolution ^27^Al MQ MAS NMR spectrum as illustrated in Fig. 5(b), where the isotropic F1 projection showed considerably improved resolution and it was clear that only two different coordinated Al species, i.e. AlO_4_ groups and AlO_6_ units, could be observed. Thus, the possible presence of alumina or any other aluminate such as five-fold coordinated Al sites^35^,^36^,^37^ can be excluded, keeping consistent with the previous XRD analysis. As a result, the structure of the YAG:Ce sample-1 can be depicted as following: YAG:Ce consists of a network of octahedral AlO_6_ and hexahedral AlO_4_ groups in which the yttrium atoms are located in the dodecahedral interstices formed by the corner sharing arrangement of the AlO_4_ and AlO_6_ polyhedra, and the incorporated Ce^3+^ ions substitute for a part of Y^3+^ ions.

### Morphology and microstructure

**Figure 6:**
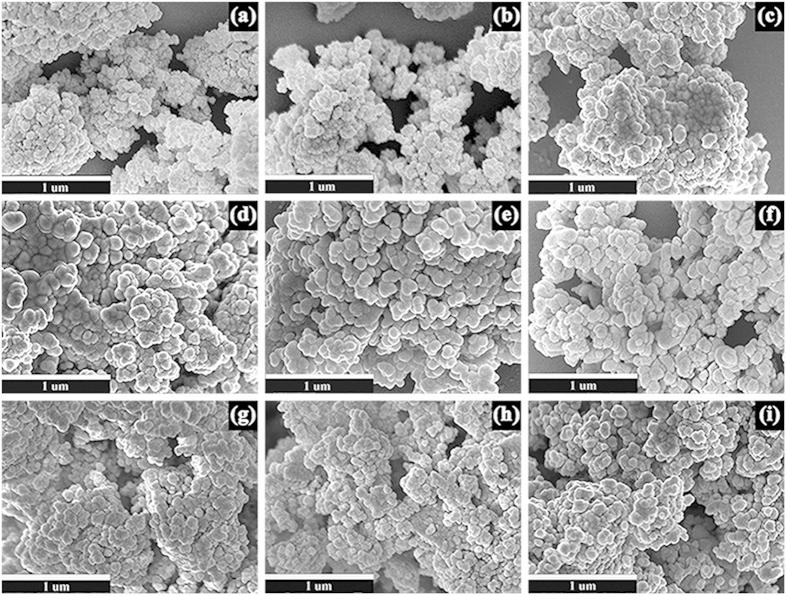
SEM images: (**a–f**) YAG:Ce sample-1 sintered at different temperatures for 3 h and (**e,g–i**) YAG:Ce samples with different Ce^3+^-doped concentrations sintered at 1030 °C for 3 h.

The SEM images of the YAG:Ce samples prepared at different temperatures are shown in Fig. 6. It is clear that these samples possess high dispersity. The mean size (*D*) values of the YAG:Ce samples measured from the SEM images are listed in Table 2. The *D* values of the YAG:Ce samples increase from ~60 to ~120 nm as the calcination temperature rises, which agrees with the XRD results^13^,^38^. In addition, Fig. 6(e,g–i) provides the SEM images of YAG:Ce samples sintered at 1030 °C with Ce^3+^-doped concentrations of 0.55, 1.10, 3.30 and 5.50 at.%. The mean size of the nanoparticles is ~95 nm and it is almost independent on the Ce^3+^ doping concentration.

**Figure 7:**
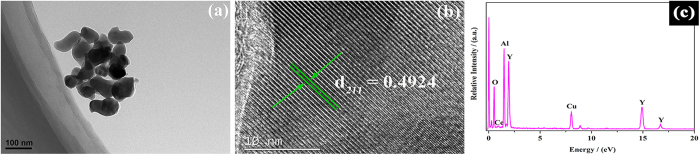
The TEM image, (**b**) HRTEM image and (**c**) EDX result of YAG:Ce sample-1 (1030 °C, 3 h).

The detailed morphology, microstructure and composition of the YAG:Ce sample-1 sintered at 1030 °C for 3 h were further analyzed by TEM, HRTEM and EDX. From Fig. 7(a), it is observed that the nanoparticles are homogeneous and well dispersed with uniform morphology. The average size of the particles is 93 nm, keeping accordance with the results from SEM characterization. Figure 7(b) shows the HRTEM image of YAG:Ce sample-1. The interplanar spacing of the lattice fringes *d*_211_ is estimated to be 0.4924 nm which is larger than the standard value of YAG (*d*_211_ = 0.4905 nm, JCPDS: 33–0040)^39^,^40^,^41^. The EDS spectrum in Fig. 7(c) confirms the presence of yttrium (Y), aluminum (Al), oxygen (O) and cerium (Ce) elements in the YAG:Ce sample-1. Except for the Cu peak resulting from the copper mesh, no other impurity can be detected in the sample.

### Chemical state at the surface

**Figure 8:**
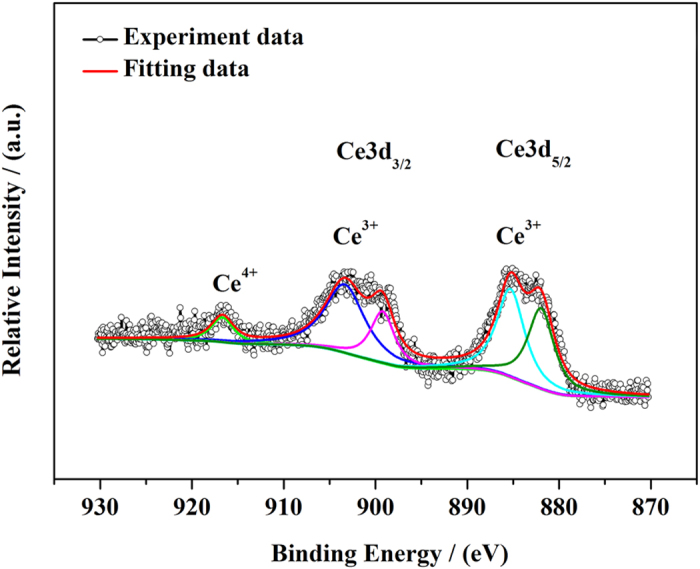
The XPS spectrum of Ce3*d* electrons in YAG:Ce sample-1 (1030 °C for 3 h).

The XPS surface measurement was applied to analyze the composition and the valence state of the incorporated Ce ions at the surface region of YAG:Ce sample-1 as shown in Fig. 8. The Ce3*d* XPS spectrum exhibits two sets of doublets (3*d*_5/2_ at 881.9 eV and 885.3 eV; 3*d*_3/2_ at 899.2 eV and 903.3 eV) which are primarily attributed to +3 oxidation state of cerium^26^,^42^,^43^,^44^. However, the appearance of another weak highest binding energy peak locating at 916.7 eV reveals the existence of a few Ce^4+^ species^44^. It is reasonable that a few cerium-(III) species were oxidized to cerium-(IV) species on the surface of the sample as it was sintered in the air.

### Photoluminescence properties and energy transfer

**Figure 9:**
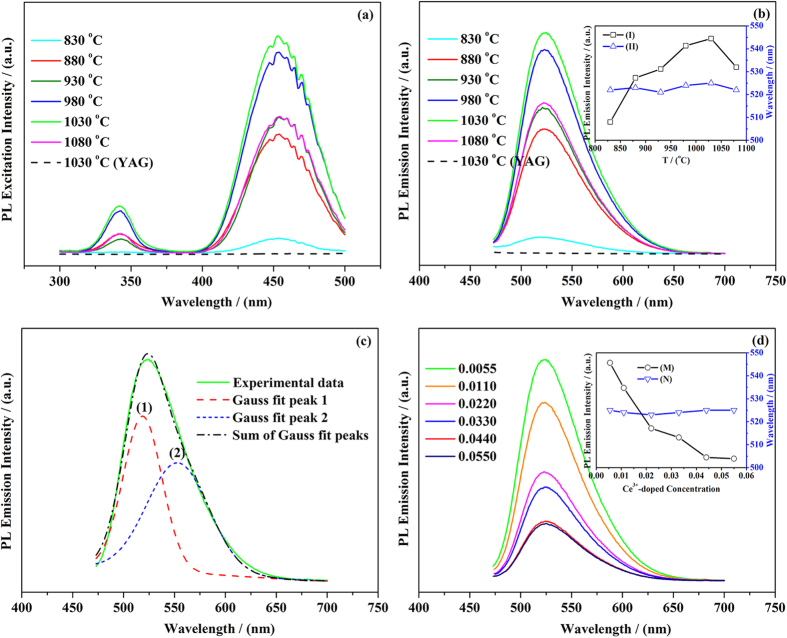
(**a**) The PLE and (**b**) PL spectra spectra of pure YAG (dash line) and YAG:Ce sample-1 sintered at different temperatures for 3 h (*λ*_em_ = 525 nm). The inset in Fig. 8(b): variation of PL intensity (I) and emission peak position (II) with the calcination temperature. (**c**) The gaussian fitted (dashed) and decomposed components (dotted) of the PL spectra of YAG:Ce sample-1 (1030 °C, 3 h). (**d**) The PL emission spectra of YAG:Ce samples with different Ce^3+^-doped concentrations (1030 °C, 3 h). Inset of Fig. 8(d): variation of PL intensity (M) and emission peak position (N) with different Ce^3+^-doped concentrations (*λ*_ex_ = 454 nm).

The PLE spectra (λ_em_ = 525 nm) of the YAG:Ce sample-1 sintered under different temperatures were measured in the wavelength range of 300–500 nm and shown in Fig. 9(a). Two prime excitation peaks are located at ~344 and ~454 nm, deriving from the 4*f*^ 1^ (^2^*F*_7/2_) → 4*f*^  0^5*d*^1^ electron transitions of Ce^3+^ ions. The shape and position of excitation peaks are nearly independent on calcination temperature. However, the PLE intensity increases with the increase of calcination temperature and exhibits the maximum excitation at 1030 °C. The excitation peak at 454 nm matches well with the blue emitting from InGaN-based LEDs. Thus, it reveals that the YAG:Ce sample-1 can efficiently absorb the blue emission and match well with InGaN LEDs.

Figure 9(b) shows the PL emission spectra (*λ*_ex_ = 454 nm) of the YAG:Ce sample-1 sintered at different temperatures. A typical broad emission band centered at ~525 nm is seen in all the samples. It is clear that the PL emission intensity strongly depends on the calcination temperature. As the calcination temperature rises from 830 to 1030 °C, the position of the PL peak shows negligible change, however, the PL emission intensity significantly increases and reaches the maximum value at 1030 °C (shown in the inset of Fig. 9(b)). The increase of emission intensity with calcination temperature is due to the improvement of crystallinity by thermal treatment, and thus more Ce^3+^ ions entering Y^3+^ lattice can be efficiently excited in YAG:Ce sample-1. Nevertheless, the emission intensity of the sample considerably decreases when the calcination temperature further increases to 1080 °C. We presume that such decrease of emission intensity is induced by cross relaxation between the adjacent Ce^3+^ ions caused by the further decreased unit cell parameters (*a*). Meanwhile, the enlarged average nanoparticles size and increased defects could cause a higher probability of nonradiative transitions as well^22^,^45^,^46^,^47^.

The strongest PL emitting curve could be fitted into two components by gaussian deconvolution, centereing at ~517 nm (peak 1) and ~558 nm (peak 2) as shown in Fig. 9(c). These two peaks correspond to the typical 5*d*^1^-4*f*^ 1^ (^2^*F*_5/2_) and 5*d*^1^-4*f*^ 1^ (^2^*F*_7/2_) transitions of Ce^3+^ ion, the energy difference between the two energy levels is ~1500 cm^−1^ because of the spin-orbital coupling in crystal-field^11^,^12^,^48^.

The PL emission spectra of the YAG:Ce samples with different Ce^3+^-doped concentrations sintered at 1030 °C for 3 h are shown in Fig. 9(d). The position of the PL peak is almost independent on the Ce^3+^ concentration, but the PL emission intensity decreases dramatically (inset of Fig. 9(d)) as the Ce^3+^ concentration increases from 0.55 to 5.50 at.%. The YAG:Ce phosphors exhibit the strongest PL emission when the Ce^3+^ ion concentration is 0.55 at.%, agreeing with the previous investigations^31^,^49^. Additionally, Zhang *et al.*^21^,^50^ studied PL intensity of YAG:Ce with lower Ce^3+^ at.% doping and found that the suitable Ce^3+^ at.% doping concentration is from 0.05 to 1 at.%. As evidenced by the previous Rietveld analysis of XRD patterns and NMR spectrum, the co-doped Ce^3+^ ion substitutes for Y^3+^ ion which is located in the dodecahedral position of YAG, and the symmetrical characteristic is *D*_2_^48^,^51^,^52^. Accompanying with the increasing Ce^3+^ doping concentration, the unit cell parameters (*a*) increased certainly, which may enlarge the distance among Ce^3+^ ions in the structure of the YAG:Ce nanocrystal. In this case, however, more nonradiation can be induced by cross relaxation of the excessive Ce^3+^ dopant. Generally, the effect of crystal field on the 4*f* state of Ce^3+^ ion is rather weak due to the shielding effect of the outer 5*p* and 6*s* electrons. Therefore, it maintains the two separate features of free ion energy levels. Whereas the 5*d* state is intensively influenced by the local crystal field surrounding the Ce^3+^ ion. Hence the *d* → *f* emission band is dependent on the local crystal field surrounding the Ce^3+^ ion^48^. Firstly, the high doping concentration hinders the substitution of Ce^3+^ ions for Y^3+^ ions owing to the large difference of ionic radius. Thus, the excessive Ce^3+^ incorporation results in more short average distance among Ce^3+^ ions and increases the probability of non-radiative transitions, thus inducing concentration quenching of Ce^3+^ ions. Secondly, the oxidation of the cerium ions (Ce^3+^→Ce^4+^) occurred near the surface can reduce the photoluminescence intensity of the Ce^3+^ activators^19^,^53^. Thirdly, for YAG phosphors with high Ce^3+^ concentration, another reason for the reduction of PL intensities is the partial absorption of excitation photons by YAG host. The competition makes less Ce^3+^ ions be excited and thus weakens the PL intensity of the YAG:Ce phosphors. To avoid the above problems, a suitable Ce^3+^ concentration and a homogeneous distribution of activators in the YAG host are important for obtaining high efficiency and brightness in the YAG:Ce system with small amount of Ce^3+^.

### The decay of photoluminescence lifetime

Fig. S1 shows the PL decay curves of YAG:Ce samples with different Ce^3+^ doping concentrations sintered at 1030 and 1080 °C. These curves can be fitted using a double-exponential function *I* = *A*_1_exp(−*t*/*τ*_1_) + *A*_2_exp(−*t*/*τ*_2_) + *I*_0_^19^,^54^,^55^, where *A*_1_ and *A*_2_ are the corresponding initial intensities of the pulse shape components and *I*_0_ is a time independent background intensity^55^. The decay includes two exponential terms, a short lifetime *τ*_1_ and a long lifetime *τ*_2_, as listed in Table 3. Among them, *τ*_1_ is likely due to the quenching of Ce^3+^ by the defects at the surface of nanoparticles, while *τ*_2_ is assigned to Ce^3+^ inside the crystalline nanoparticles^19^,^56^. The large specific surface area of the nanoparticles has profound effects on the photoluminescence properties because of the partial oxidation of activator Ce^3+^ to Ce^4+^ on the surface. Since more surface defects formed due to the increase of specific surface area, the PL lifetime of Ce^3+^-doped YAG was shortened than that of the ideal YAG:Ce crystal^55^,^56^.

As shown in Table 3, both *τ*_1_ and *τ*_2_ of the YAG:Ce sample-1 are slightly shortened when the calcination temperature increased from 1030 to 1080 °C. The slight variation of PL lifetime could be attributed to the further oxidation of cerium ion (Ce^3+^→Ce^4+^) by the excessive calcination, which is consistent with the result of PL characterization (shown in Fig. 9(b)). Therefore, an appropriate calcination temperature is favorable to achieving strong PL emission and decreasing the PL decay. Moreover, it can be found in Table 3 that the PL lifetimes (both *τ*_1_ and *τ*_2_) of YAG:Ce sample-6 are much shorter than that of the YAG:Ce sample-1. Generally, the PL lifetime *τ* can be written as *τ* = (*γ*_r_ +*γ*_nr_)^−1^, where *γ*_r_ is the decay rate of radiative process and *γ*_nr_ is the rate of non-radiative process^54^,^56^. In the Ce^3+^-doped YAG phosphors, the substitution of Ce^3+^ ions for Y^3+^ ions results in the creation of structural defects such as surface Ce^4+^, non-luminescent aggregates of Ce^3+^ ions, Y_Al_ antisite defects and so on^57^, which could act as the quenching center of luminescence^56^. Consequently, the increase of Ce^3+^ concentration would enhance the non-radiative transition. According to the above equation, larger *γ*_nr_ of the YAG:Ce sample-6 gives rise to a shorter lifetime *τ* relative to YAG:Ce sample-1. Therefore, it is seen from Fig. 9(d) that the superfluous Ce^3+^ incorporation leads to PL quenching and decreases the PL intensity, i.e. increases the PL decay considerably. From the above investigation, we clarified the relationship between local structure of Ce^3+^ ions in YAG:Ce phosphors and photoluminescence properties. Meanwhile, we obtained a good YAG:Ce photoluminescence material by precisely adjusting the Ce^3+^ concentration and the calcination temperature.

## Conclusion

Nanocrystalline YAG:Ce phosphors with good dispersity were successfully prepared by sol-gel method. The photoluminescence properties of the YAG:Ce nanophosphors was adjusted by changing the calcination temperature and the Ce^3+^ concentration. It was found that the photoluminescence emission intensity of YAG:Ce nanophosphor was enhanced with the increase of calcination temperature in the range of 830–1030 °C, while dramatically decreased with the increase of Ce^3+^-doped concentration. The strongest photoluminescence emission band centering at 525 nm was obtained in YAG:Ce_0.0055_ nanophosphors sintered at 1030 °C for 3 h. Moreover, the studies on fluorescence lifetime suggested that the decay contained two exponential terms, i.e. the short lifetime τ_1_ (~28 ns) and the long lifetime τ_2_ (~94 ns). The fluorescence lifetime was not sensitive to the calcination temperature, but decreased markedly with the increase of Ce^3+^-doped concentration. This study not only provides a good YAG:Ce photoluminescence material but also benefits the clarification of the relationship between local structure of incorporated Ce^3+^ and photoluminescence properties.

## Experimental Procedure

### Preparation of (Y_1−*x*_Ce_*x*_)_3_Al_5_O_12_ (YAG:Ce_*x*_) phosphors

All reagents are of analytical grade and used without further purification. The precursors were synthesized by sol-gel method using Y(NO_3_)_3_·6H_2_O, Ce(NO_3_)_3_·6H_2_O and Al(NO_3_)_3_·9H_2_O as cation resource, citric acid (CA) and ethylene glycol (EG) as chelating agents with a ratio of Cations: CA: EG = 2: 2: 1. The Y(NO_3_)_3_·6H_2_O and Al(NO_3_)_3_·9H_2_O were firstly dissolved in 120 mL distilled water with stoichiometric ratio of 3 ×(1−*x*): 5 with *x* varying from 0.0055 to 0.0550, and then CA and EG were added into the above solution. The solution was continuously stirred at 50 °C to evaporate excess water and accelerate the polyesterification reaction. The gels were heated in oil-bath at 100 °C for 24 h. Finally, the precursors were ground and sintered at 830, 880, 930, 980, 1030 and 1080 °C respectively for 3 h in air. The obtained YAG:Ce_*x*_ phosphors were denoted as sample-1, sample-2, sample-3, sample-4, sample-5 and sample-6 corresponding to *x* values of 0.0055, 0.0110, 0.0220, 0.0330, 0.0440, 0.0550).

### Material characterization

The thermal process was recorded by thermogravimetry analysis (TGA) and differential scanning calorimetry (DSC) at the heating rate of 10 °C·min^−1^ on a Netzsch STA 449F^3^ instrument. The FT-IR spectra of powders were measured on a Thorlabs Nicolet Nexus 6700 infrared spectrophotometer. The crystal structure of powders were characterized by X-ray diffraction (XRD) on a Rigaku D/max-2500 diffractometer using Cu-*K*_*α*_ radiation filtered by graphite with the experimental parameters of 40 kV, 200 mA, and 6° min^−1^. Then Rietveld were performed on the collected data by using Fullprof 2K software to get the lattice parameters. The ^27^Al solid state NMR spectra of the products were acquired at 104.2 MHz on a Bruker Avance III HD 400M NMR spectrometer (9.4 T) equipped a 4 mm MAS NMR probe with a spinning rate of 10 kHz. The morphology was observed by a JEOL JSM-7500F scanning electron microscope (SEM). The microstructure and composition were measured by a JEOL JEM-2100F transmission electron microscope (TEM) equipped with an Oxford energy dispersive spectrometer (EDS). The chemical state were analyzed via Thermo Scientific X-ray photoelectron spectroscopy (XPS) using a monochromatized Al-*K*_*α*_ X-ray as the excitation source and choosing O 1*s* at a binding energy of 530.5 eV as the reference line. The photoluminescence emission (PL) and photoluminescence excitation (PLE) spectra of sample powders were obtained on a Hitachi F-4600 fluorescence spectrophotometer using a 150 W Xenon short-arc lamp as excitation source (wavelength range: 200–900 nm, excitation slit: 2.5 nm, emission slit: 2.5 nm, PMT voltage: 700 V). The fluorescence lifetimes (FLS) were measured by an Edinburgh FLS920 phosphorimeter using a 450 W xenon lamp. All the measurements were implemented at room temperature.

## Additional Information

**How to cite this article**: He, X. *et al.* Effects of local structure of Ce^3+^ ions on luminescent properties of Y_3_Al_5_O_12_:Ce nanoparticles. *Sci. Rep.* **6**, 22238; doi: 10.1038/srep22238 (2016).

## Supplementary Material

Supplementary Information

## Figures and Tables

**Table 1:** Crystallographic data with atomic parameters of YAG:Ce_*x*_ samples.

**Atomic parameters of YAG:Ce**_***x***_ **sample-1**							
Atoms	Wyckoff	x/*a*	y/*b*	z/*c*	Cell parameter (Å)	Cell volume (Å)^3^	Mean size (Å)
Al 1	16a	0.00000	0.00000	0.00000	12.0354	1743.336	208
Al 2	24d	0.37500	0.00000	0.25000			
Y 1	24c	0.12500	0.00000	0.25000			
Ce 1	24c	0.12500	0.00000	0.25000			
O 1	96 h	−0.02985	0.05056	0.14878			
Al 1	16a	0.00000	0.00000	0.00000	12.0220	1737.523	238
Al 2	24d	0.37500	0.00000	0.25000			
Y 1	24c	0.12500	0.00000	0.25000			
Ce 1	24c	0.12500	0.00000	0.25000			
O 1	96 h	−0.03139	0.05148	0.14915			
Al 1	16a	0.00000	0.00000	0.00000	12.0198	1736.557	243
Al 2	24d	0.37500	0.00000	0.25000			
Y 1	24c	0.12500	0.00000	0.25000			
Ce 1	24c	0.12500	0.00000	0.25000			
O 1	96 h	−0.02998	0.05117	0.14989			
Al 1	16a	0.00000	0.00000	0.00000	12.0079	1731.408	310
Al 2	24 d	0.37500	0.00000	0.25000			
Y 1	24c	0.12500	0.00000	0.25000			
Ce 1	24c	0.12500	0.00000	0.25000			
O 1	96 h	−0.03161	0.05207	0.14913			
Al 1	16a	0.00000	0.00000	0.00000	12.0093	1732.026	270
Al 2	24d	0.37500	0.00000	0.25000			
Y 1	24c	0.12500	0.00000	0.25000			
Ce 1	24c	0.12500	0.00000	0.25000			
O 1	96 h	−0.03177	0.05011	0.14924			
Al 1	16a	0.00000	0.00000	0.00000	12.0197	1736.522	268
Al 2	24d	0.37500	0.00000	0.25000			
Y 1	24c	0.12500	0.00000	0.25000			
Ce 1	24c	0.12500	0.00000	0.25000			
O 1	96 h	−0.03259	0.04875	0.14842			

**Table 2:** The mean size (*D*) values of YAG:Ce samples.

Samples	a	b	c	d	e	f	g	h	i
T (^o^C)	830	880	930	980	1030	1080	1030	1030	1030
Ce^3+^(at.%)	0.55	0.55	0.55	0.55	0.55	0.55	1.10	3.30	5.50
*D* (nm)	61	66	75	84	95	118	91	90	93

**Table 3:** Decay times of YAG:Ce phosphors.

Sample	Temperature	Lifetime *τ*_1_ (ns)	Lifetime *τ*_2_ (ns)
YAG:Ce sample-1	1030	27.8	94.0
YAG:Ce sample-1	1080	26.5	91.1
YAG:Ce sample-6	1030	18.0	52.3
