# Supplementary material for: Effects of local structure of Ce3+ ions on luminescent properties of Y3Al5O12:Ce nanoparticles
Source: Sci Rep. 2016 Mar 3;6:22238. doi: 10.1038/srep22238 (PMC4776235; doi:10.1038/srep22238)
Supplement: Supplementary Information [file srep22238-s1.pdf]

**Supplementary Information (SI)**

# Effects of local structure of $Ce^{3+}$ ions on luminescent properties of $Y_3Al_5O_{12}$:Ce nanoparticles

Xiaowu He, Xiaofang Liu*, Rongfeng Li, Bai Yang, Kaili Yu, Min Zeng, Ronghai Yu*

School of Materials Science and Engineering, Beihang University, Beijing, 100191, P. R. China

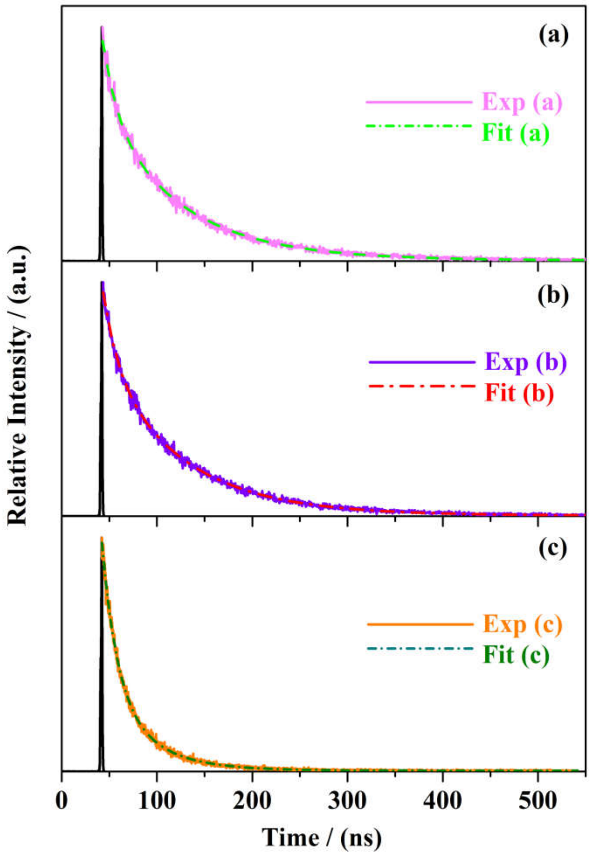


Fig. S1 (a) PL decays of YAG:Ce sample-1 (1030 °C, 3 h), (b) PL decay of YAG:Ce sample-1 (1080 °C, 3 h) and (c) PL decay of YAG:Ce sample-6 (1030 °C, 3 h) ($\lambda_{ex}$ = 454 nm, $\lambda_{ex}$ = 525 nm). The photoluminescence decay curves show that the fluorescence lifetimes of YAG:Ce samples with different $Ce^{3+}$ doping concentrations sintered at 1030 and 1080 °C.
